# P029: Regional differences in Clostridium difficile infections (CDI) in relation to fluoroquinolone (FQ) and proton pump inhibitor (PPI) use, Finland, 2008-2011

**DOI:** 10.1186/2047-2994-2-S1-P29

**Published:** 2013-06-20

**Authors:** M Kanerva, J Ollgren, T Voipio, S Mentula, O Lyytikäinen

**Affiliations:** 1 Infectious Diseases, Helsnki University Central Hospital, Helsinki, Finland; 2 National Institute for Health and Welfare, Helsinki, Finland; 3 Finnish Medical Agency, Helsinki, Finland

## Introduction

In Finland, incidence of CDI increased during 2002-2006, shown by the population-based analysis of hospital discharge diagnoses, a trend similar to that in the US. In 2008, toxin-positive *C. difficile* became a notifiable disease. During the first 3 years of surveillance, the annual incidence decreased from 111 to 90/100,000 population, but rose up to 100 in 2011. The epidemic situation and trends differed regionally. Both PPI and FQ use have been associated with increased risk of CDI.

## Objectives

To study whether the use of antibiotics, FQs and PPIs were associated with regional differences in CDI rates.

## Methods

Data on CDI incidence during 2008-2011 in 21 hospital districts (HD) was obtained from the National Infectious Disease Registry and consumption of antibiotics and PPIs from the Finnish Medical Agency. The availability of molecular methods in diagnostics was obtained by a laboratory survey and data on FQ resistant ribotypes from the national reference laboratory. Negative binomial regression model was performed to assess the impact of different antibiotics, PPIs, the presence of ribotype 027 CD and the use of molecular diagnostic methods in the respective HD on CDI incidence. Variable selection in the model was done by using Akaike information criteria (AIC).

## Results

The level of FQ use was stable during 2008-2011 (although it had doubled during 1990-2000). The use of PPI increased 50% during 2008-2011. FQ use was strongly associated with the CDI incidence, and there was a trend between PPI use and the CDI incidence in different HDs. The presence of molecular methods (including PCR) or the knowledge of ribotype 027 being detected in the HD was not associated with the CDI incidence. According to AIC, in the final multivariable model, we only included FQ use. The incidence rate ratio for FQs was 2.45 (95%CI, 1.58-3.79; p<0.001).

## Conclusion

FQ use was an important risk factor for CDI, as shown previously. However, the use has stabilized during the last few years. In the national guidelines, FQs are included in the choices to treat urinary tract infections but not respiratory tract infections. The increasing use of PPIs is of note.

## Disclosure of interest

None declared

